# A Deep Neural Network for Interpreting Wearable Electrocardiogram Data in Atrial Fibrillation: Prospective Observational Diagnostic Accuracy Study

**DOI:** 10.2196/82475

**Published:** 2026-04-23

**Authors:** Olli A Rantula, Jukka A Lipponen, Jari Halonen, Helena Jäntti, Tuomas T Rissanen, Mika P Tarvainen, Noora S Naukkarinen, Eemu-Samuli Seljola, Onni E Santala, Jagdeep Sedha, Tero J Martikainen, Juha E K Hartikainen

**Affiliations:** 1 School of Medicine, Faculty of Health Sciences, University of Eastern Finland, Yliopistonranta 1, PO BOX 1627, Kuopio, 70211, Finland, 358 0294451111; 2 Doctoral School, Faculty of Health Sciences, University of Eastern Finland, Kuopio, Finland; 3 Heart Center, Kuopio University Hospital, Kuopio, Finland; 4 Department of Technical Physics, Faculty of Science and Forestry, University of Eastern Finland, Kuopio, Finland; 5 Department of Anaesthesiology and Intensive Care, North Karelia Central Hospital, Joensuu, Finland; 6 Heart Center, North Karelia Central Hospital, Joensuu, Finland; 7 Faculty of Medicine, University of Helsinki, Helsinki, Finland; 8 Conservative Ward Department, Mikkeli Central Hospital, Mikkeli, Finland; 9 Anesthesiology and Intensive Care, Kuopio University Hospital, Kuopio, Finland; 10 Department of Prehospital Emergency Care, Acute Services, Kuopio University Hospital, Kuopio, Finland

**Keywords:** atrial fibrillation, single-lead electrocardiogram, ambulatory monitoring, automatic rhythm detection, artificial intelligence, deep neural network

## Abstract

**Background:**

Atrial fibrillation (AF) and atrial flutter (AFL) are common arrhythmias associated with the risk of ischemic stroke, which can be reduced with anticoagulation therapy. Thus, early diagnosis of AF and AFL is essential. However, diagnosis may be challenging due to the paroxysmal and asymptomatic nature of these arrhythmias.

**Objective:**

Current diagnostic workflows involve time-consuming and resource-intensive manual review of noisy signals and prolonged recordings. We evaluated a mobile system that combines a wireless wearable single-lead chest strap electrocardiogram (ECG) and a novel deep neural network (DNN)–based artificial intelligence (AI) method for detecting AF/AFL episodes, AF/AFL burden, and rhythm change and estimated the delay in the detection of rhythm change from AF/AFL to sinus rhythm. We also assessed the rhythm classification performance.

**Methods:**

A total of 116 patients with recent-onset AF or AFL undergoing cardioversion were monitored using a mobile single-lead chest strap ECG system. Simultaneously, a 3-lead Holter ECG served as the reference. The DNN-based AI analyzed the single-lead chest strap ECG data to detect AF/AFL, non-AF/AFLrhythm, and noninterpretable segments, as well as to estimate AF/AFL burden and detect rhythm change. Performance metrics included sensitivity, specificity, positive predictive value, negative predictive value, and intraclass correlation coefficient for AF and AFL burden estimation.

**Results:**

The sensitivity and specificity for detecting AF/AFL were 91.9% (204.9/223.0 h) and 99.6% (242.4/243.5 h), respectively. The sensitivity for detecting AF was 96.2% (191.5/199.0 h), whereas it was 55.8% (13.4/24.0 h) for detecting AFL. The positive predictive value and negative predictive value for AF/AFL detection were 99.5% (204.9/206.0 h) and 93.1% (242.4/260.5 h), respectively. The intraclass correlation coefficient between the AF/AFL burden estimated by the DNN-based AI method and that derived from the physician-interpreted reference ECG was 0.96 (95% CI: 0.94‐0.97; *P*<.001). Rhythm change detection occurred within 1 minute in most cases.

**Conclusions:**

The mobile single-lead chest strap ECG system powered by a DNN-based AI algorithm demonstrated strong performance in detecting AF, estimating AF burden, and recognizing rhythm change to sinus rhythm. This AI-driven approach enables automated and accurate rhythm analysis, supporting clinical decision-making. Further validation in real-world ambulatory settings is warranted.

## Introduction

Atrial fibrillation (AF) is the most common cardiac tachyarrhythmia worldwide. It is characterized by uncoordinated electrical activation of the atria and irregular rhythm. AF is often accompanied by atrial flutter (AFL). Both AF and AFL reduce quality of life and are associated with significant morbidity and mortality [1-6]. The most severe complication related to AF is ischemic stroke. At least two-thirds of AF-related strokes can be prevented with proper anticoagulation treatment if AF is diagnosed early enough [1,6-10]. AFL is also associated with increased stroke risk, although the risk is somewhat lower than the risk related to AF [11]. Therefore, a similar risk evaluation and anticoagulation policy is recommended for AF and AFL [8]. With an aging population, the prevalence of AF and AFL is increasing, and screening for AF in patients at high risk for stroke is recommended [8,12-15]. The European Society of Cardiology guidelines recommend opportunistic screening for AF in individuals aged over 65 years [8]. The diagnosis of AF and AFL requires a high-quality electrocardiogram (ECG) recording which must be interpreted by a physician [8]. However, the diagnosis of AF and AFL is challenging, as they are often paroxysmal and frequently asymptomatic. Traditional 12-lead and ambulatory ECGs are limited by their time frame and are far from ideal prolonged everyday monitoring [8].

The diagnosis of AF and AFL in current clinical practice typically prompts the initiation of anticoagulation therapy if specific risk factors for stroke are present [8,9]. In addition to the presence of AF and AFL, AF and AFL burden contribute to the stroke risk. AF and AFL burden refers to the cumulative time spent in AF or AFL during a monitoring period. It reflects both the frequency and duration of AF episodes. This measure has been proposed as one of the metrics for guiding personalized AF and AFL management [8].

Several types of wearable sensors are available and capable of capturing ECG signals. These may be used for arrhythmia detection in paroxysmal AF and AFL. However, longer recordings generate more data, and interpretation represents a substantial workload for physicians, increasing resource use and health care costs.

Particularly, increasing the duration of recordings require the use of automatic and reliable data analytics to reduce manual effort and minimize resource waste. Various automated algorithms are available for analyzing extended ECG data, and several studies have evaluated their effectiveness in arrhythmia detection [16-22]. However, data from wearable devices, which are usually equipped with dry electrodes, often contain extensive noise and artifacts, which can negatively impact detection accuracy and further increase the workload required to analyze the recordings. The development of advanced artificial intelligence (AI) has provided new tools for automated data analysis.

In this study, we used a mobile single-lead chest strap ECG system that combines a wireless, mobile phone–connected consumer-grade ECG device with an automatic deep neural network (DNN)–based artificial intelligence (AI) method. The DNN-based method was originally developed for analyzing 3-lead ambulatory ECG recordings to detect AF and AFL.

The aim of this study was to evaluate the diagnostic performance of this DNN-based method when applied to wearable single-lead ECG data, including its ability to detect AF/AFL episodes; estimate AF/AFL burden; identify rhythm changes and associated detection delays; and classify recordings into the AF/AFL, non-AF/AFL, and noninterpretable categories.

## Methods

### Study Population and Participation Criteria

This study is part of a larger research project Wearables and Biomarkers in Detecting Atrial Fibrillation (WB-AF). It is registered in the ClinicalTrials database (NCT04917653).

Data collection was conducted at the emergency care department of Kuopio University Hospital. A total of 291 volunteer patients were screened between June 2022 and July 2023. The inclusion criteria were recent-onset (<48 h) AF or AFL confirmed by a 12-lead ECG, age ≥18 years, and being scheduled for cardioversion during the index treatment period at the emergency department. The type of cardioversion included but was not limited to electrical synchronized defibrillation, antiarrhythmic medication, and spontaneous rhythm change. Successful rhythm change was not a requirement for inclusion.

The exclusion criteria were BMI ≥35 kg/m^2^, a cardiac pacemaker, or a medical condition requiring immediate treatment. Patients with BMI ≥35 kg/m² were excluded for practical reasons related to device fit and stability, as the chest strap ECG sensor may not reliably fit or remain in place in individuals with severe obesity. This exclusion was applied to ensure proper functioning of the recording setup.

Initially, 127 patients were recruited in the study. Eleven patients were excluded due to technical issues related to data acquisition. In 9 cases, data were missing because of a bug or instability in the measurement application. In 2 cases, problems related to device use led to complete corruption of the recorded data. The final study population comprised 116 patients with AF or AFL. Of these, 109 patients successfully resumed sinus rhythm (SR) and had both AF/AFL and SR data available, while 7 patients had only AF/AFL data due to unsuccessful cardioversion.

Clinical data were extracted from medical records and patient interviews. Symptoms reported during ECG recordings were documented using a structured questionnaire designed for this study. The CHA₂DS₂-VA score (congestive heart failure, hypertension, age ≥75 years, diabetes, prior stroke or transient ischemic attack, vascular disease, age 65-74 years) was used to estimate the risk of stroke. This scoring system is in line with clinical guidelines and accounts for several risk factors. In this system, 2 points each are assigned for age ≥75 years and a history of stroke or transient ischemic attack, and 1 point each is assigned for age between 65 and 74 years, congestive heart failure, hypertension, diabetes mellitus, and vascular disease. In accordance with clinical guidelines, a score of ≥2 indicates an elevated stroke risk and most likely indicates the need for anticoagulant therapy [8].

### Mobile Single-Lead Chest Strap ECG System and Automated DNN-Based AI Method for Rhythm Detection

Our mobile single-lead chest strap ECG system consisted of 3 main components: a wearable single-lead chest strap ECG sensor, a dedicated mobile phone application for data collection, and a DNN-based AI method for analytics. The ECG sensor, along with an elastic chest-worn belt designed for consumer use (Movesense Medical, Movesense Ltd.), recorded single-lead ECG signals at a sampling rate of 256 Hz. Data were transmitted wirelessly via Bluetooth to a mobile phone, which ran an in-house–developed mobile application responsible for data collection. The data were then forwarded to a DNN-based AI method operating on a dedicated computing platform.

In this study, we used a DNN model based on a residual network architecture developed in our research group. A similar residual network has been successfully applied to ECG arrhythmia classification tasks in earlier work, including the study by Hannun et al [23].

The DNN-based AI method was trained using ECG data from a total of 15,816 recordings obtained from 15,242 patients across 7 heterogeneous datasets collected using different clinical devices. Of these, 10,248 recordings originated from publicly available annotated AF/AFL datasets, and 5568 recordings were derived from locally collected clinical datasets from Kuopio University Hospital and from our previous studies. The training data consisted of multilead ECG recordings, including standard 12-lead ECGs and ambulatory ECG recordings with durations ranging from 30 minutes to 24 hours (Holter-type recordings), reflecting typical real-world clinical conditions. Recordings were acquired using different ECG systems with sampling rates ranging from 125 to 500 Hz and most commonly included leads I, II, and V3, although lead configurations varied slightly between datasets. Across the training dataset, 3180 patients had AF or AFL, while the remaining recordings represented non-AF/AFL rhythms. The training dataset did not include any single-lead ECG recordings.

The model processes raw ECG input in 10-second segments, each consisting of 3 input channels sampled at 125 Hz. In the original model architecture, these channels correspond to 3 distinct ECG leads. Because the chest strap device used in this study provides only a single-lead ECG signal, the same waveform was replicated across all 3 input channels when applying the model to chest strap recordings. This design choice was made to preserve compatibility with the original, previously validated 3-channel network architecture without modifying the model structure or retraining the network.

No additional patient-related information was required, enabling fully automated analysis based solely on the ECG waveform. Each 10-second segment was classified into 1 of 3 categories: AF/AFL; non-AF/AFL, including SR; or noninterpretable due to poor signal quality.

The model produces an independent rhythm classification for each 10-second segment. To improve clinical relevance and reduce short detections, the model output was subsequently postprocessed to account for temporal continuity of rhythms. Specifically, consecutive 10-second segments classified as AF/AFL were combined into continuous episodes. AF/AFL detections shorter than 30 seconds (fewer than 3 consecutive AF/AFL-positive 10-second segments) were excluded to align with commonly used guideline-based definitions that require at least 30 seconds of continuous single-lead ECG for AF diagnosis [8]. Closely spaced AF/AFL segments separated by short gaps were merged into unified episodes. Performance metrics were calculated on the basis of the total duration of correctly and incorrectly classified 10-second segments and are reported as hours correctly classified divided by total hours (h/h). AF burden was defined as the total duration of AF/AFL during the ECG recording period.

### Reference for Rhythm Detection and Quality Analysis

Written informed consent was obtained from all patients after eligibility was confirmed in the emergency department. Immediately after consent was obtained, patients were equipped with both the mobile single-lead chest strap ECG device and a 3-lead ambulatory Holter ECG device, and continuous ECG monitoring was initiated.

ECG monitoring continued throughout the treatment period, including during cardioversion and subsequent observation, and the devices were removed prior to hospital discharge. All analyses of algorithm performance were performed retrospectively after data collection was complete.

The concurrently recorded 3-lead Holter ECG was used as the reference standard. Reference ECG data were recorded at a sampling frequency of 250 Hz using an ambulatory Holter device (Faros 360, Bittium), capturing leads I, II, and V3. Synchronization between the single-lead and 3-lead recordings was initially based on timestamps and was manually verified by aligning the recordings to the documented time of cardioversion.

All reference ECG recordings were manually interpreted by an experienced physician trained in ECG interpretation. The physician annotated rhythm categories (AF, AFL, and SR) and the timing of cardioversion. The same physician also visually assessed the single-lead chest strap ECG recordings for signal quality. Periods of at least 10 seconds considered noninterpretable due to poor signal quality were labeled as noninterpretable, whereas shorter noisy periods were assumed to represent the same rhythm as that observed immediately before and after the segment. These physician-labeled annotations served as the reference for evaluating the performance of the DNN-based method in detecting noninterpretable segments.

### Statistical Methods

Rhythm detection performance was evaluated using both time-based and episode-based (patient-level) approaches.

In the time-based analysis, the mobile single-lead chest strap ECG data were analyzed as consecutive 10-second intervals. Each interval classified by the DNN-based AI method was compared with the corresponding reference rhythm derived from the 3-lead Holter ECG. A classification was considered correct if the rhythm category (AF/AFL, non-AF/AFL, or noninterpretable) matched the reference for the same time interval.

In the episode-based analysis, ECG recordings were evaluated at the patient level to assess whether AF or AFL was detected during reference-confirmed AF/AFL episodes. A patient was considered correctly identified as AF/AFL-positive if the DNN-based method detected at least 1 AF/AFL segment within a reference-confirmed AF/AFL episode. Performance was assessed against the reference using sensitivity, specificity, positive predictive value (PPV), negative predictive value (NPV), and Youden index. For episode-based AF/AFL detection, the Youden index was calculated to summarize the overall balance between sensitivity and specificity. Sensitivity was also calculated separately for AF and AFL.

Performance metrics included sensitivity, specificity, PPV, NPV, *F*_1_-score, and Cohen kappa. For proportion-based metrics (sensitivity, specificity, PPV, and NPV), 95% CIs were calculated using Wilson score intervals. For *F*_1_-scores, 95% CIs were estimated using nonparametric bootstrap resampling (2000 iterations). Cohen kappa was used to assess the agreement between rhythm categories (AF/AFL, non-AF/AFL, or noninterpretable) assigned by the DNN-based AI method and the reference.

The AF/AFL burden estimated by the DNN-based method was compared with the reference AF/AFL burden derived from the 3-lead ECG using Bland-Altman analysis, reporting the mean difference (bias) and 95% limits of agreement (mean difference ± 1.96 SD). Agreement was further assessed using the intraclass correlation coefficient (ICC) based on a 2-way mixed-effects model with absolute agreement and single measurements (ICC model 3.1). Bland-Altman and scatter plots were used for visualization [24].

Delay in rhythm change detection was defined as the time difference between the first SR segment identified in the reference 3-lead ECG after conversion from AF/AFL and the time point at which the DNN-based method first classified the rhythm as SR in the single-lead ECG after postprocessing. Delays were calculated for each AF/AFL-to-SR transition event and summarized descriptively. A 2-sided significance level of *P*<.05 was used.

### Ethical Considerations

This study received approval from the Ethics Committee of the Northern Savo Hospital District (Approval number: 2061/2020). Prior to enrollment, all participants were provided with detailed written information about the study procedures, and written informed consent was obtained from each participant. Participation was voluntary, and participants had the right to withdraw from the study at any time. This study complied with the ethical principles set forth in the Declaration of Helsinki for research involving human participants.

All data were anonymized prior to analysis, and no identifiable personal information was included in the dataset. Data were handled and stored in accordance with applicable data protection regulations. Participants did not receive any financial compensation for participation in this study.

## Results

### Study Population

**Figure 1.**
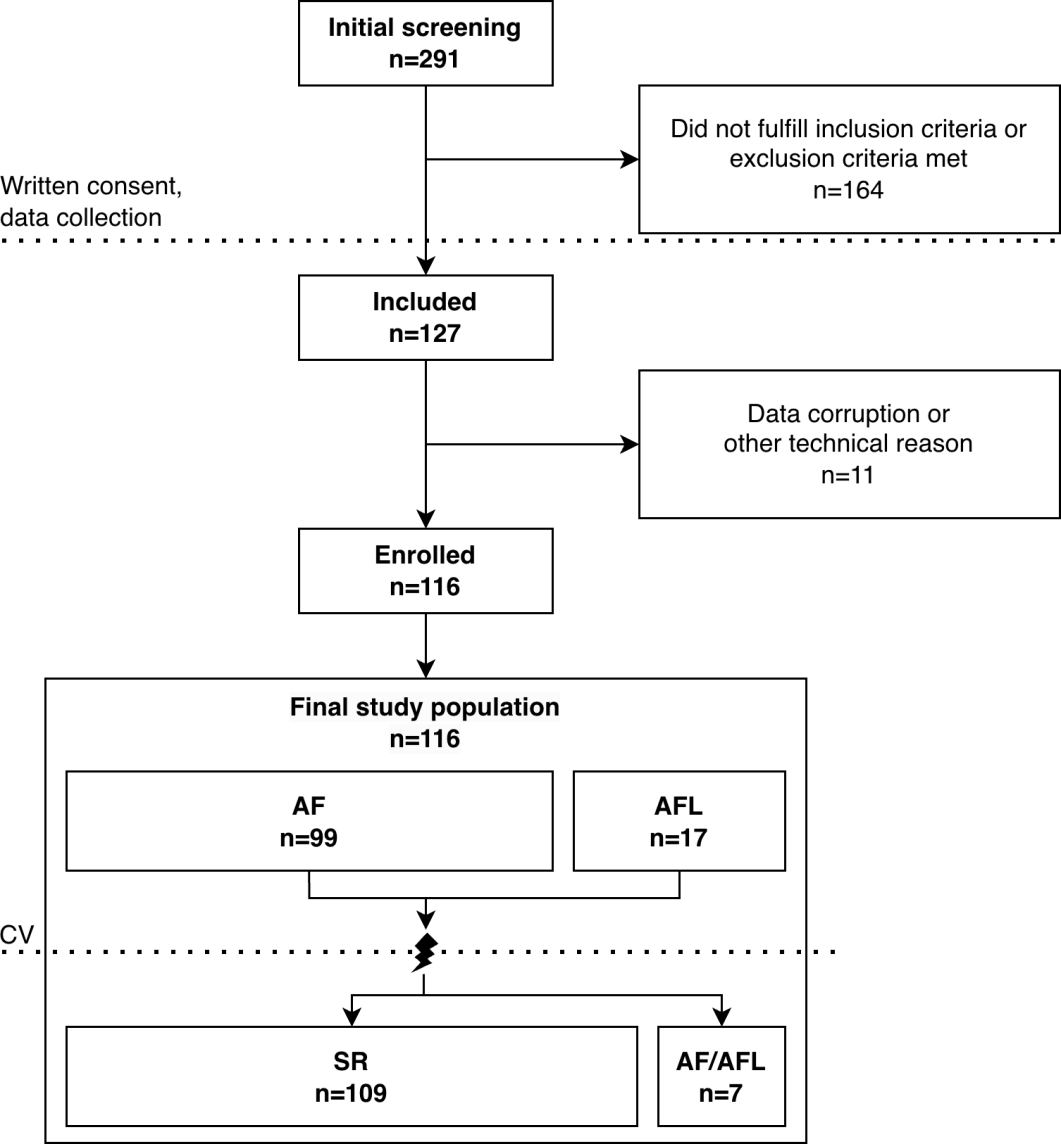
Data collection flowchart. AF, atrial fibrillation; AFL, atrial flutter; CV, cardioversion; SR, sinus rhythm.

The final study population comprised data collected from 116 patients diagnosed with AF (n=99) or AFL (n=17) who were scheduled for SR restoration. The data collection flowchart is presented in Figure 1. SR was achieved in 109 patients. The mean age was 65.1 (SD 11.4) years (range 24-90 years), the mean BMI 27.5 (SD 4.2) kg/m² (range 18.8-34.9 kg/m²), and 60.3% (70/116) of the patients were male (Table 1). Hypertension was the most prevalent comorbidity present in 51.7% (60/116) patients, 90.5% (105/116) had a prior history of AF or AFL, and 9.5% (11/116) presented with first-diagnosed AF/AFL. The most frequently reported symptoms prior to hospital admission were palpitations (88.8%, 103/116), fatigue (50%, 58/116), and decline of general condition (44.8%, 52/116). The CHA_2_DS_2_-VA score was 0, 1, and ≥2 for 27 (23.3%), 32 (27.6%), and 57 (49.1%) of the 116 patients, respectively.

**Table 1.** Patient demographics (N=116).

	Value
Characteristic	
Age (years), mean (SD)	65.1 (11.4)
Weight (kg), mean (SD)	83.0 (15.9)
Height (cm), mean (SD)	173.6 (9.4)
BMI (kg/m^2^), mean (SD)	27.5 (4.2)
Male sex, n (%)	70 (60.3)
Recording	
Overall duration (min), mean (SD)	241.2 (93.6)
AF^a^/AFL^b^ duration (min), mean (SD)	122.0 (87.1)
SR^c^ duration (min), mean (SD)	119.2 (46.6)
Medical history	
Previous AF diagnosis, n (%)	105 (90.5)
Hypertension, n (%)	60 (51.7)
Coronary artery disease, n (%)	18 (15.5)
Structural heart disease^d^, n (%)	17 (14.6)
Diabetes, n (%)	16 (13.8)
Other arrhythmia^e^, n (%)	8 (6.9)
Prior cardiac surgery, n (%)	8 (6.9)
Congestive heart failure, n (%)	2 (1.7)
Medication	
Beta blocker, n (%)	92 (79.3)
Anticoagulation therapy, n (%)	74 (63.8)
Flecainide, n (%)	15 (12.9)
Amiodarone, n (%)	4 (3.4)
Digoxin, n (%)	1 (0.9)
Symptoms during to hospital admission	
Palpitation, n (%)	103 (88.8)
Fatigue, n (%)	58 (50)
General condition decline, n (%)	52 (44.8)
Respiratory distress, n (%)	23 (19.8)
Chest pain, n (%)	15 (12.9)
Thromboembolic risk	
CHA_2_DS_2_VA^f^=0, n (%)	27 (23.3)
CHA_2_DS_2_VA=1, n (%)	32 (27.6)
CHA_2_DS_2_VA≥2, n (%)	57 (49.1)

a AF: atrial fibrillation.

b AFL: atrial flutter.

c SR: sinus rhythm.

d Presence of any physician-documented structural heart disease in the medical records.

e Previously diagnosed arrhythmia excluding atrial fibrillation or atrial flutter.

f The CHA₂DS₂-VA score estimates stroke risk in patients with AF/AFL and includes the following components: congestive heart failure (1 point), hypertension (1 point), age 65‐75 years (1 point), diabetes mellitus (1 point), vascular disease (1 point), prior stroke or transient ischemic attack (2 points), and age >75 years (2 points).

### ECG Data Characteristics, Quality, and Rhythm Classification

A total of 466.3 hours of concurrent single-lead chest strap ECG and reference 3-lead ECG data were collected, which included 223.0 hours (47.8%) of AF/AFL, consisting of 199.0 hours (42.7%) of AF and 24.0 hours (5.1%) of AFL as defined by the reference. Additionally, 219.0 hours (46.9%) of data represented SR based on the reference ECG. The physician identified 24.5 hours (5.3%) of single-lead chest strap ECG data as noninterpretable (Table 2).

**Table 2.** Confusion matrix for rhythm classification by the deep neural network (DNN)–based artificial intelligence method compared with the reference 3-lead Holter electrocardiogram^a^.

DNN prediction	AF^b^ (Holter)^c^	AFL^d^ (Holter)	Non-AF/AFL (Holter)	Noninterpretable (physician)
AF/AFL	191.5 (41.1)	13.4 (2.9)	0.06 (0.0)	1.0 (0.2)
Non-AF/AFL	2.6 (0.6)	10.2 (2.2)	214.1 (45.9)	1.9 (0.4)
Noninterpretable	4.9 (1)	0.4 (0.1)	4.8 (1)	21.6 (4.6)

a Values are reported as number of hours (%) of total recording time (ie, 466.3 h).

b AF: atrial fibrillation.

c Holter: Holter monitor.

d AFL: atrial flutter.

The time-based performance of the DNN-based AI method in detecting AF/AFL, non-AF/AFL, and noninterpretable episodes is presented in the confusion matrix (Table 2), providing an overview of the classification results. The DNN-based AI method correctly classified 204.9 of 466.3 hours (43.9%) as AF/AFL. Misclassification into this category was minimal, comprising 0.06 of 466.3 hours (0%) of non-AF/AFL rhythm and 1.0 of 466.3 hours (0.2%) of noninterpretable data (Table 2). For SR, the DNN-based AI method correctly identified 214.1 of 466.3 hours (45.9%) as non-AF/AFL but misclassified 12.8 of 466.3 hours (2.7%) of actual AF/AFL and 1.9 of 466.3 hours (0.4%) of actual noninterpretable data into this category.

Additionally, the DNN-based AI method classified 31.7 of 466.3 hours (6.8%) of the data as noninterpretable, whereas the physician classified 24.5 of 466.3 hours (5.3%) as noninterpretable on the basis of visual assessment of the single-lead ECG (Table 2). Of the algorithm-labeled noninterpretable data, 21.6 of 466.3 hours (4.6%) were also considered noninterpretable by the physician.

The difference was due to different signal quality thresholds and handling of short noisy segments between the algorithm and the physician. The physician labeled noisy periods of at least 10 seconds as noninterpretable, whereas shorter noisy segments were assumed to represent the surrounding rhythm. The DNN-based method evaluated each 10-second segment independently and could classify individual segments as noninterpretable even when adjacent segments were interpretable.

The DNN-based method assigned a rhythm label of AF/AFL and non-AF/AFL to 1.0 (0.2%) and 1.9 (0.4%) of 466.3 hours, respectively, which the physician considered noninterpretable due to poor signal quality. Conversely, the algorithm classified 4.9 hours (1.1%) during AF, 4.8 hours (1%) during SR, and 0.4 hours (0.1%) during AFL as noninterpretable despite the reference rhythm being known from the Holter ECG (Table 2). Importantly, this difference did not meaningfully affect the AF/AFL burden estimation, as reflected by the high agreement between methods (ICC 0.96).

**Figure 2.**
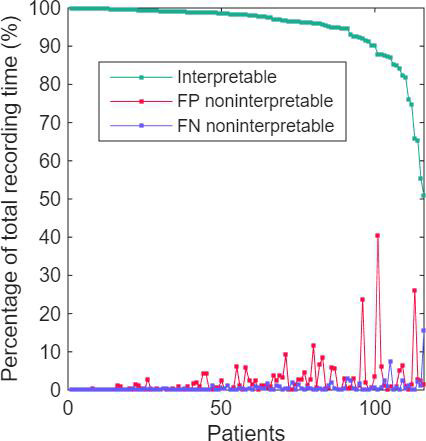
Proportion of interpretable single-lead electrocardiogram (ECG) data and classification errors per patient. Each data point represents 1 patient (N=116). Patients are ordered from left to right according to decreasing proportion of ECG data considered interpretable on the basis of physician review of the single-lead chest strap recordings. The y-axis shows the percentage of total recording time per patient. The green curve represents the proportion of data considered interpretable according to physician review. Red points indicate false-positive noninterpretable classifications (segments classified as noninterpretable by the DNN but considered interpretable by physician review), and blue points indicate false-negative noninterpretable classifications (segments classified as interpretable by the DNN but considered noninterpretable by physician review). FP, false positive; FN, false negative.

For most participants, the proportion of single-lead chest strap ECG data considered interpretable on the basis of physician review exceeded 95% of the total recording time, and false-positive and false-negative noninterpretable classifications were generally low (Figure 2). In 3 patients, more than 20% of the recorded data were falsely classified as noninterpretable by the DNN-based AI method. In these patients, the total recording duration ranged from 1.0 to 5.0 hours, of which 0.4 to 2.2 hours were falsely classified as noninterpretable.

### Performance of the DNN-Based AI Method for Time-Based Rhythm Detection

The performance of the DNN-based AI method in detecting rhythm categories showed an overall accuracy of 94.4% (440.3/466.3 h). Cohen kappa was 0.90, indicating excellent agreement between the DNN-based AI method and the reference.

For AF/AFL detection, the DNN-based AI method achieved a time-based sensitivity of 91.9% (204.9/223.0 h) and a specificity of 99.6% (242.4/243.5 h), with a PPV of 99.5% (204.9/206.0 h) and an NPV of 93.1% (242.4/260.5 h), yielding an *F*_1_-score of 0.96 (Table 3). More specifically, the DNN-based AI method achieved a time-based detection sensitivity of 96.2% (191.5/199.0 h) for AF and 55.8% (13.4/24.0 h) for AFL, indicating stronger performance in identifying AF than AFL. For SR detection, the DNN-based AI method achieved a sensitivity of 97.8% (214.1/219.0 h) and a specificity of 94.1% (232.8/247.5 h), with a PPV of 93.6% (214.1/228.8 h) and an NPV of 97.9% (232.8/237.7 h), yielding an *F*_1_-score of 0.96 (Table 3).

The performance of the mobile single-lead chest strap ECG system compared with the physician’s interpretation for detecting noninterpretable cases was lower, with a time-based sensitivity of 88.2% (21.6/24.5 h) and a specificity of 97.7% (431.9/442.0 h; Table 3). This category had a PPV of 68.1% (21.6/31.7 h) and an NPV of 99.3% (431.9/434.8 h), yielding an *F*_1_-score of 0.77. These results highlight the challenges in detecting and categorizing ambiguous or unclear cases. Detection results for the noninterpretable data category of the DNN-based AI method are presented in Figure 2.

**Table 3.** Performance metrics of the deep neural network (DNN)–based artificial intelligence method for rhythm detection from single-lead chest strap electrocardiogram data.

Metric^a^	AF^b^/AFL^c^	Non-AF/AFL	Noninterpretable
Sensitivity	204.9/223.0 (91.9, 95% CI^d^ 91.6‐92.2)	214.1/219.0 (97.8 95% CI 97.6‐97.9)	21.6/24.5 (88.2, 95% CI 87.2‐88.8)
Sensitivity for AF^e^	191.5/199.0 (96.2, 95% CI 96‐96.5)	—^f^	—
Sensitivity for AFL^e^	13.4/24.0 (55.8, 95% CI 54‐57.6)	—	—
Specificity	242.4/243.5 (99.6, 95% CI 99.5‐99.6)	232.8/247.5 (94.1, 95% CI 93.9‐94.4)	431.9/442.0 (97.7, 95% CI 97.6‐97.8)
PPV^g^	204.9/206.0 (99.5, 95% CI 99.4‐99.5)	214.1/228.8 (93.6, 95% CI 93.3‐93.9)	21.6/31.7 (68.1, 95% CI 66.6‐69.6)
NPV^h^	242.4/260.5 (93.1, 95% CI 92.8‐93.3)	232.8/237.7 (97.9, 95% CI 97.7‐98.1)	431.9/434.8 (99.3, 95% CI 99.3‐99.4)
*F*_1_-score^i^	0.96 (95% CI 0.95‐0.96)	0.96 (95% CI 0.95‐0.96)	0.77 (95% CI 0.76‐0.78)
Proportion of data	222.9/466.3 (47.8)	219.0/466.3 (46.9)	24.5/466.3 (5.3)

a Performance metrics are based on 10-second segments and reported as correctly classified hours divided by total hours (%).

b AF: atrial fibrillation.

c AFL: atrial flutter.

d 95% CIs for sensitivity, specificity, PPV, and NPV were calculated using Wilson score intervals.

e Because the DNN outputs a combined AF/AFL class, AF- and AFL-specific results can be reported only as sensitivities (proportion of AF or AFL time classified as AF/AFL).

f Not applicable.

g PPV: positive predictive value.

h NPV: negative predictive value.

i 95% CIs for *F*_1_-scores were estimated using bootstrap resampling.

### Performance of the DNN-Based AI Method for Detection of AF/AFL Episodes, Rhythm Change, and AF/AFL Burden

**Figure 3.**
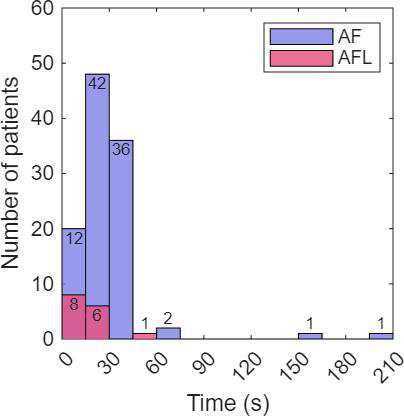
Distribution of delay in rhythm change recognition from AF/AFL to sinus rhythm following cardioversion or spontaneous rhythm change. AF, atrial fibrillation; AFL, atrial flutter.

For episode detection, the sensitivity and specificity of AF/AFL detection were 97.4% (113/116) and 98.2% (107/109), respectively, with a PPV of 98.3% (113/115) and an NPV of 97.3% (107/110). When calculated solely for AF cases (excluding AFL), the sensitivity was 100% (100/100) for AF episode detection and 81% (13/16) for AFL episode detection (Table 4). The Youden index for AF/AFL detection was 0.96. The delay in rhythm change detection after rhythm change was less than 1 minute in most cases (Figure 3).

**Table 4.** Atrial fibrillation (AF)/atrial flutter (AFL) episode detection and AF/AFL burden estimation.

Metric	Value
Episode-based confusion matrix for DNN^a^ AF/AFL detection	
True Positive AF/AFL episode detection	113
False Positive AF/AFL episode detection	2
False Negative AF/AFL episode detection	3
True Negative AF/AFL episode detection	107
Episode-based performance metrics^b^	
Sensitivity for AF/AFL, episodes/episodes (%)	113/116 (97.4, 95% CI 92.7‐99.1)
Specificity for AF/AFL, episodes/episodes (%)	107/109 (98.2, 95% CI 93.6‐99.5)
PPV^c^ for AF/AFL, episodes/episodes (%)	113/115 (98.3, 95% CI 93.9‐99.5)
NPV^d^ for AF/AFL, episodes/episodes (%)	107/110 (97.3, 95% CI 92.3‐99.1)
Sensitivity for AF, episodes/episodes (%)	100/100 (100, 95% CI 96.3‐100)
Sensitivity for AFL, episodes/episodes (%)	13/16 (81, 95% CI 57‐93)
Youden index for AF/AFL	0.96
Mean AF/AFL burden estimated by DNN (h), mean (SD)	1.8 (1.5)
Mean AF/AFL burden by Holter electrocardiogram (h), mean (SD)	2.0 (1.5)
ICC^e^ for AF/AFL burden	0.96 (95% CI 0.94‐0.97; *P*<.001)

a DNN: deep neural network.

b Episode-based performance metrics are derived from the 2×2 confusion matrix (TP, FP, FN, and TN) shown in the table and are reported as episodes correctly classified divided by the total number of episodes (%).

c PPV: positive predictive value.

d NPV: negative predictive value.

e ICC: intraclass correlation coefficient.

**Figure 4.**
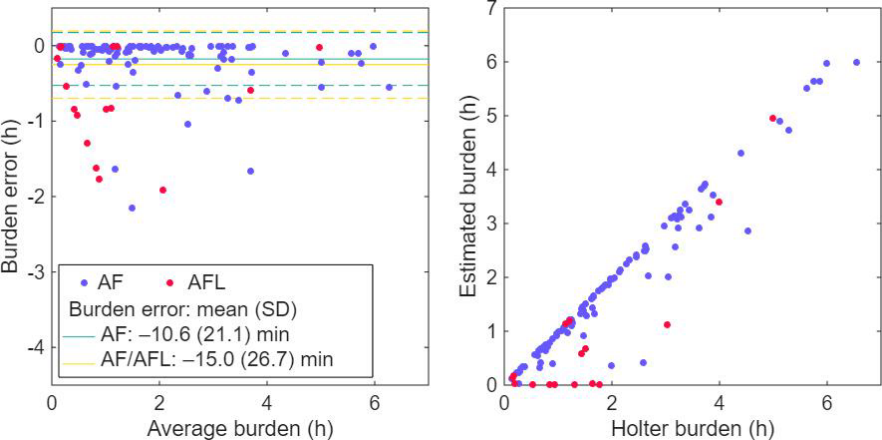
Bland-Altman plot and scatter plot of atrial fibrillation (AF) and atrial flutter (AFL) burden estimated from single-lead chest strap electrocardiogram (ECG) using a deep neural network–based artificial intelligence method compared with the reference 3-lead Holter ECG. The left panel shows the Bland-Altman plot, and the right panel shows the correlation between the estimated and reference AF/AFL burden.

The mean AF/AFL burden estimated by the mobile single-lead chest strap ECG system was 1.8 (SD 1.5) hours, whereas the corresponding burden derived from the reference ECG was 2.0 (SD 1.5) hours (Table 4). The mean difference in AF/AFL burden was −15.0 (SD 26.7) minutes, and for AF alone, it was −10.6 (SD 21.1) minutes. The Bland-Altman analysis demonstrated a mean bias of −0.25 hours for AF/AFL burden estimation, with 95% limits of agreement ranging from −1.12 to 0.62 hours. The Bland-Altman plot and scatter plot comparing the AF/AFL burden estimates between the DNN-based method and the reference are shown in Figure 4. Agreement between the methods was high, with an ICC of 0.96 (95% CI 0.94‐0.97; *P*<.001) (Table 4). Visual inspection of the Bland-Altman plot did not suggest the presence of proportional bias across the range of AF/AFL burden values.

## Discussion

### Principal Findings

The main finding of this study was that the DNN-based AI method applied to data obtained from a mobile chest strap ECG system demonstrated high diagnostic accuracy for detecting AF episodes, identifying rhythm changes from AF to SR, and estimating AF burden, with adequate sensitivity and specificity and strong agreement with the reference method. The mobile single-lead chest strap ECG system detected AF episodes more accurately than AFL episodes.

Our findings demonstrate high performance in the use of wearable devices and AI algorithms for AF detection, comparable to or exceeding results reported in previous studies [16-21,25-31]. For instance, the BASEL Wearable Study demonstrated that various consumer-grade smart devices achieved reasonable sensitivity (up to 85%) and specificity (up to 75%) for AF detection, although the rate of inconclusive recordings by the automated detection method was relatively high (ranging from 17% to 26%, depending on the device). However, manual review indicated that a majority of the tracings were interpretable [25]. Similarly, Pan et al [32] showed that using longer, 7-day recordings, smartwatch-based single-lead ECG can detect AF episodes with a duration of >5 minutes with a sensitivity of 89.7% and specificity of 67.4%. Moreover, the systematic review and meta-analysis by Manetas-Stavrakakis et al [31], which evaluated the diagnostic accuracy of AI-based technologies for AF detection, reported pooled sensitivity and specificity values of 92.3% and 96.2% for single-lead ECG, respectively.

Recent clinical studies further support the feasibility of artificial intelligence–based atrial fibrillation detection using single-lead ECG. De Guio et al [33] demonstrated enhanced detection of AF using a cloud-based AI platform applied to single-lead ECG recordings, highlighting the clinical applicability of AI-assisted rhythm analysis in simplified ECG configurations. Zhao et al [34] also developed a temporal convolutional network model for AF detection using single-lead ECG and reported high diagnostic accuracy, supporting the applicability of advanced neural network architectures to wearable ECG data.

In comparison, our study demonstrated even higher sensitivity (96.2%) for AF detection and comparable sensitivity (91.9%) and specificity (99.5%) for AF/AFL detection using the mobile single-lead chest strap ECG system, further encouraging the potential of consumer-grade devices in clinical settings in providing more information for diagnostics about AF estimation and the burden of AF.

The DNN-based AI method was trained using ECG data from a diverse patient population worldwide, incorporating various device types and lead configurations, including 3- and 4-lead Holter monitors and 12-lead ECG recordings. The model was evaluated using a single-lead ECG configuration that was not represented in the training data, as the chest strap lead does not correspond directly to any of the training leads. This setup allowed assessment of whether the model had learned features that generalize beyond specific lead configurations. The observed performance suggests that the model captures AF/AFL-related features that are transferable across different electrode placements, which may support its applicability across heterogeneous wearable ECG devices.

However, AFL detection was less reliable than AF detection, as reflected by the substantially lower sensitivity for AFL. This finding is clinically relevant and likely reflects the inherent limitations of single-lead ECG morphology in recognizing atrial flutter. Unlike AF, which is characterized by highly irregular RR intervals, AFL often presents with more regular ventricular response and subtle atrial activity patterns that can be difficult to distinguish from SR when only a single lead is available. In addition, the DNN-based model used in this study was primarily trained on 3-lead ECG data, which provide richer spatial information on atrial activity. The transition from a 3-lead training environment to a single-lead wearable setting may therefore limit the ability to identify characteristic flutter wave morphology.

Furthermore, single-lead wearable ECG recordings are more susceptible to motion artifacts and noise, which may affect the detection of AFL compared to AF and contribute to a higher proportion of noninterpretable segments. Similar challenges have been reported in previous studies, where AI models trained on multilead ECG data demonstrated reduced performance when applied to single-lead recordings, particularly for arrhythmias with overlapping waveform characteristics [35,36]. From a clinical perspective, these findings suggest that while the system performs robustly for AF detection and AF burden estimation, caution is warranted when interpreting potential AFL cases. In situations where AFL is suspected or diagnostic uncertainty remains, confirmation using higher-quality or multilead ECG recordings may be necessary. These results also highlight the need for further development of AI models specifically optimized for single-lead ECG data to improve AFL detection in wearable monitoring settings.

The high sensitivity and specificity for AF detection suggest that the DNN-based AI method is robust and reliable for identifying AF episodes when data quality is sufficient. Accurate detection of rhythm changes between SR and AF is essential for identifying paroxysmal AF. In AF screening, the primary aim is to detect AF episodes and rhythm changes from SR to AF. Additionally, for assessing AF burden, it is important to detect both AF episodes and rhythm changes in both directions, from SR to AF and from AF to SR. In this study, only rhythm changes from AF to SR were detected due to the study protocol. However, given the high time-based detection performance with an accuracy of 94.4%, we assume that the DNN-based AI method is also capable of detecting rhythm changes from SR to AF.

AF burden is recognized in current clinical guidelines as an important predictor of stroke risk and a relevant factor in clinical decision-making [8]. Therefore, estimating AF burden from single-lead ECG data is particularly valuable for the continuous monitoring and management of AF and may serve as a complementary tool in clinical evaluation. Pan et al [32] showed sufficient correlation regarding AF burden between single-lead ECG and reference Holter ECG with an ICC of 0.927 over a 7-day monitoring period. Moreover, Hennings et al [37] demonstrated a high correlation (Pearson correlation coefficient 0.998) between an AI-based tool used to detect AF burden and manual assessment of AF burden in 7-day Holter ECG recording, with minimal negative bias of AF burden. Similarly, in our study, the ICC was 0.96, with a low rhythm change recognition time and low negative bias. In most cases, rhythm changes were detected immediately or at least within 1 minute after cardioversion. The high level of agreement between the AF burden measurements obtained using the mobile single-lead chest strap ECG system and the reference Holter ECG reinforces the validity of this approach for single-lead ECG usage. Notably, we used a dry-electrode ECG instead of a Holter monitor. This method is particularly suitable for long-term monitoring and is more comfortable than Holter monitors with potentially irritating wet electrodes. Moreover, the use of ECG allows for the diagnosis of AF according to current clinical guidelines [8].

### Study Limitations

Despite the promising results, there are some limitations to consider. The study population was limited to patients with recent-onset AF or AFL who presented to the emergency department, which does not represent the broader population with paroxysmal or persistent AF/AFL with fluctuating rhythm. Patients were permitted to move freely during the study recordings. However, while hospitalized, they were primarily lying in the supine position, which affects the conclusions regarding the use of the devices in real-world settings. Additionally, studies involving a more diverse patient population and longer monitoring periods in real-world ambulatory settings are needed to validate the generalizability and long-term performance of this approach.

### Conclusions

The high sensitivity and specificity for AF detection and strong correlation for AF burden suggest that the mobile chest strap ECG system, in which a DNN-based AI method is used with single-lead chest strap ECG, is promising for identifying AF episodes, rhythm change, and AF burden. A majority of the data was analyzable, and no complaints or discomfort regarding the wearables or study devices were reported.

Our study indicates the potential of consumer-grade single-lead ECG devices combined with an advanced detection algorithm for the detection and monitoring of AF/AFL, independent of the specific ECG device. This approach offers promising complementary tools for the continuous monitoring, diagnosis, and personalized management of AF and AFL.

## Abbreviations

AF: atrial fibrillation
AFL: atrial flutter
AI: artificial intelligence
CHA₂DS₂-VA score: congestive heart failure, hypertension, age ≥75 years, diabetes, prior stroke or transient ischemic attack, vascular disease, age 65-74 years
DNN: deep neural network
ECG: electrocardiogram
ICC: intraclass correlation coefficient
KUH: Kuopio University Hospital
NPV: negative predictive value
PPV: positive predictive value
SR: sinus rhythm
